# Supplementary material for: Collaborative intelligence in AI: Evaluating the performance of a council of AIs on the USMLE
Source: PLOS Digit Health. 2025 Oct 9;4(10):e0000787. doi: 10.1371/journal.pdig.0000787 (PMC12510544; doi:10.1371/journal.pdig.0000787)
Supplement: S2 File — (ZIP) [file pdig.0000787.s002.zip › S2 Data Raw data files containing unprocessed question inputs and LLM outputs including transcripts of deliberations/STEP3_Question-92-1_Type-MC-NJ.docx]

**Facilitator** **[Round 1]:** A 25-year-old man comes to the office because he has had a ""coating"" on his tongue during the past 2 weeks and has had an unintentional 4.5-kg (10-lb) weight loss during the past 6 months. He has not had oral pain or difficulty swallowing. Medical history is unremarkable and he takes no medications. He has smoked one pack of cigarettes daily for the past 5 years and he drinks two to three beers weekly. He is sexually active with two male partners and does not consistently use condoms. BMI is 25kg/m². Vital signs are temperature 37.1°C (98.8°F), pulse 83/min, respirations 16/min, and blood pressure 114/74 mm Hg. Oral examination shows a white plaque on the lateral aspect of the tongue bilaterally; an attempt to scrape the lesion from the tongue is unsuccessful. Anterior and posterior cervical lymph nodes are enlarged bilaterally but are mobile and not tender to palpation. The remainder of the physical examination discloses no abnormalities. A rapid HIV test is positive. Results of other laboratory studies are shown: Blood CD4+ T-lymphocyte count - 128/mm³ Hematocrit - 36% Hemoglobin - 12.0 g/dL WBC - 1400/mm³ Neutrophils, segmented - 79% Eosinophils - 3% Lymphocytes - 11% Monocytes - 7% Platelet count - 124,000/mm³ Which is of the following is the most appropriate clinical intervention at this time? (A) Antiretroviral therapy (B) Liquid nitrogen therapy applied to the tongue lesions (C) Micafungin therapy (D) Surgical excision of the tongue lesions (E) Systemic chemotherapy with liposomal doxorubicin

**AI_B:** (1) RESTATING THE QUESTION: The question is asking about the most appropriate next step in the clinical management of a 25-year-old man who has a coating on his tongue along with cervical lymphadenopathy and a positive HIV test. The patient's CD4 count is found to be low, indicative of late-stage HIV infection, and he shows several risk factors for HIV, including unprotected sex with multiple partners and smoking habits. (2) PLANNING: The question assumes that the most suitable intervention will address the most pressing and critical disease manifestation, which includes the positive HIV status, low CD4 count, and oral findings. We must evaluate which of these should be addressed primarily based on urgency and potential harm. However, it's reasonable to suggest the introduction of antiretroviral therapy to suppress HIV viral replication and disease progression may be a prioritized assumption given the risk of fatality associated with untreated HIV. (3) CLINICAL REASONING: The coating on the patient's tongue and low CD4 count suggest a possible opportunistic infection. The white plaques, which cannot be scraped off, along with positive HIV status, suggest a probable diagnosis of oral hairy leukoplakia, a condition that typically occurs in severely immunocompromised patients. It's caused by Epstein-Barr virus and often associated with a low CD4 count. However, while oral lesions require attention, it's paramount to address his late-stage HIV disease, potentially leading to AIDS-defining conditions if untreated. Prompt initiation of antiretroviral therapy (ART) is crucial as it can slow or halt disease progression and restore immune function, thus preventing other opportunistic infections linked to an impoverished immune system. The other options, such as liquid nitrogen therapy, micafungin therapy, and surgical excision, primarily deal with the tongue lesion but are not essential at this time. Similarly, systemic chemotherapy with liposomal doxorubicin is not indicated as there's no sign of neoplastic disease requiring such aggressive treatment. (4) CONCLUDING: Using these clinical reasoning steps, it becomes evident that addressing the underlying cause for the patient's immunodeficient state, i.e., starting antiretroviral therapy for HIV, is a priority. (5) RESPONSE: Therefore, the most appropriate clinical intervention at this time is to start antiretroviral therapy (Option A).

**AI_E:** (1) RESTATING THE QUESTION: This case presents a 25-year-old man who has a white coating on his tongue and unintentional weight loss. He reports no pain in the mouth or difficulty swallowing, smokes a pack of cigarettes daily, drinks moderately, and is sexually active with multiple male partners without consistent condom use. His body mass index is within the normal range, and his vital signs are stable. His oral exam reveals a persistent white plaque on his tongue, while his lymph nodes are enlarged but not tender. Aside from these findings, his physical examination is normal. However, he tests positive for HIV, and his bloodwork suggests a potential condition related to his immune system, with a low CD4+ T-lymphocyte count and low white blood cell count. The question asks for the best immediate intervention. (2) PLANNING: Primary assumption is that the patient's oral manifestations and significantly reduced CD4+ T-lymphocyte count are HIV-related findings. White coating on his tongue may be oral candidiasis. An alternative assumption could be another condition causing the symptoms, but the given scenario, patient's high-risk sexual behaviours, and his positive HIV test all strongly imply a scenario of advance HIV disease. (3) CLINICAL REASONING: HIV is known to inflict damage on the immune system by gradually depleting CD4+ T-lymphocytes, which are crucial for maintaining an effective immune response. As a result, patients with AIDS (HIV disease with CD4+ count less than 200/mm³, as in this patient) are prone to various opportunistic infections, oral candidiasis being one of the most common. Clinically, oral candidiasis may present as a white plaque on the tongue that cannot be scraped off. Considering other options, liquid nitrogen therapy and surgical excision are not typical treatments for oral candidiasis. Micafungin is an antifungal drug but it's mostly reserved for treatment of resistant cases and invasive candidiasis, not the oral thrush that this patient has. Systemic chemotherapy is not appropriate as there are no signs of a systemic cancer diagnosis. (4) CONCLUDING: Therefore, the most immediate therapeutic need is to initiate antiretroviral therapy (ART) considering his AIDS diagnosis and to treat the oral candidiasis (presumably with a more standard first-line agent). ART will boost the patient's immune response over time, thus helping to prevent further opportunistic infections. (5) RESPONSE: The most appropriate clinical intervention at this time for this patient is to begin antiretroviral therapy (option A).

**AI_D:** RESTATING THE QUESTION: The patient presented is a 25-year-old male with a medical history of unprotected sexual activity with multiple male partners. He's reported unintentional weight loss over a period and a recent appearance of a coating on his tongue that cannot be scraped away. His vital signs are within normal parameters, but his lymph nodes are noticeably enlarged. A rapid HIV test has returned positive, and his blood tests show a significant reduction in his CD4+ T-lymphocyte count, suggesting immunodeficiency. The query is about the best clinical intervention to undertake in this particular scenario, with options ranging from antiretroviral therapy, specific treatments for the tongue lesions, or systemic chemotherapy. PLANNING: The first assumption would be that the tongue lesions and weight loss are indicators of AIDS-related complications, given the positive HIV test and decreased CD4+ T-lymphocyte count. Such complications could include oral candidiasis or Kaposi’s sarcoma. The inability to scrape away the lesion could suggest oral hairy leukoplakia, another AIDS-related condition. However, the systemic effects of untreated HIV infection should also be considered. Accordingly, the priority should be to manage his HIV. CLINICAL REASONING: When connecting the patient's symptoms and medical history to his diagnostic results, several key points arise. His high-risk sexual behavior and positive HIV test, together with a CD4+ T-lymphocyte count below 200/mm³, meet the criteria for AIDS diagnosis according to CDC guidelines. His tongue lesions, which can’t be scraped off, suggest oral hairy leukoplakia, a condition often seen in people with HIV/AIDS. Oral hairy leukoplakia is usually a painless, asymptomatic lesion caused by Epstein-Barr virus reactivation due to immunodeficiency. His weight loss and lymphadenopathy (enlarged lymph nodes) are further indications of advanced disease. The patient's low CD4+ count and presence of characteristic manifestations necessitate immediate initiation of antiretroviral therapy (ART), regardless of symptoms or the absence thereof, under current recommendations. CONCLUDING: Comparing the treatment options, surgical excision or systemic chemotherapy would not address the underlying cause. Liquid nitrogen and micafungin treat other conditions like warts or fungal infections, not typically present in this scenario. Therefore, the optimal choice is to initiate antiretroviral therapy, which will act against HIV replication, slow disease progression, and improve immune function. RESPONSE: The most appropriate clinical intervention at this time is to start antiretroviral therapy (Option A), as it addresses the underlying cause of the patient’s symptoms and will contribute to managing his HIV infection effectively.

**AI_C:** RESTATING THE QUESTION: A 25-year-old male presents with a two-week history of a white "coating" on his tongue and a six-month history of unexplained weight loss. He has no history of oral pain nor difficulty swallowing, as well as a non-distinct medical history. He smokes and drinks occasionally, has multiple sexual partners and inconsistent condom use. His BMI is within range, and his vital signs are stable. On oral examination, there are immovable white patches on both sides of his tongue. Upon physical examination, mobile, non-tender lymph nodes are noted in the anterior and posterior cervical regions. His HIV test came out positive. His lab results display a decreased CD4+ T-lymphocyte count, a reduced WBC count, and a lower than normal platelet count. The question here is what would be the best clinical intervention at this point. PLANNING: Based on his clinical signs, symptoms, lifestyle choices, physical examination, and laboratory results, we have assumptions regarding opportunistic infections, HIV progression, and AIDS. The primary assumption being that the man's symptoms and lab results may be due to an opportunistic infection resulting from HIV infection. Notably, the presence of immovable white plaques on the tongue that cannot be scraped off could suggest a fungal infection, particularly oral candidiasis, usually occurring in immunocompromised patients. This hypothesis is further reinforced by the patient's significantly decreased CD4+ T-lymphocyte count, confirming an impaired immune response. CLINICAL REASONING: We know that the progression of HIV infection can lead to impaired T-lymphocyte function, making the host susceptible to opportunistic infections, including fungal infections. Oral candidiasis often presents as white patches in the mouth that can't be scraped off. The presence of such lesions, in conjunction with the patient's low CD4+ T-lymphocyte count (128/mm³ is significantly below the normal range of 500-1500/mm³), weight loss, and positive HIV test, indicates a progression of the disease to Acquired Immunodeficiency Syndrome (AIDS). In this case, antiretroviral therapy (ART) is typically administered to control the progression of the disease. CONCLUDING: To address the HIV progression and the opportunistic fungal infection, the most appropriate intervention would be antiretroviral therapy (ART). ART works by suppressing the HIV virus and halting the progression of the disease, thereby improving the patient's immune function and mitigating the effects of opportunistic infections. RESPONSE: Therefore, the recommended course of action at this stage would be (A) Antiretroviral therapy. However, it's essential to note that further counselling and monitoring should follow to ensure medication adherence and manage any side effects. Treatment of specific opportunistic infections (like oral candidiasis) should also be addressed.

**AI_A:** 1) RESTATING THE QUESTION: In this scenario, we are managing a 25-year-old male patient who has noted white plaque-like developments on his tongue over the past two weeks and has experienced unintentional weight loss in the last six months. He doesn’t have any significant medical history. He practices risk behaviors such as smoking, moderate alcohol intake, and unprotected sexual activity with multiple partners. Notably, his HIV test returned positive, and further tests showed a low CD4+ count, as well as other hematological abnormalities. Several treatment approaches are proposed, including antiretroviral therapy, liquid nitrogen for the oral lesions, micafungin therapy, surgical excision of the oral lesions, or systemic chemotherapy with liposomal doxorubicin. Our task is to identify the most appropriate intervention at this moment. 2) PLANNING: The key information in this scenario includes the white plaque on the tongue, unintentional weight loss, and the patient's HIV-positive status with a low CD4+ cell count. The white plaques on the tongue that cannot be scraped off, combined with the low CD4+ count and positive HIV status, lead us to suspect oral hairy leukoplakia or oral candidiasis, opportunistic infections commonly seen in immune-compromised patients, such as those with HIV/AIDS. Although systemic antiretroviral therapy will eventually be needed to manage the HIV infection, immediate intervention should focus on treating possible opportunistic infections for immediate symptomatic relief. Consequently, the first assumption is that micafungin might be justified. 3) CLINICAL REASONING: Oral candidiasis is one of the most common HIV-related opportunistic infections and can be identified by white plaques that can typically be scraped off, contrasting with our case. Oral hairy leukoplakia, caused by Epstein-Barr virus (EBV), presents as white plaques typically on the lateral aspects of the tongue that cannot be scraped off, aligning better with our case. Micafungin, an antifungal, would be appropriate if we were dealing primarily with oral candidiasis. However, oral hairy leukoplakia, which is caused by a viral infection, doesn’t typically require antiviral therapy, unless severe, and doesn't respond to antifungal treatments. Therefore, though micafungin was the first assumption, after reviewing the clinical markers, it doesn't seem the most appropriate choice. 4) CONCLUDING: Therefore, the correct decision should focus directly on the HIV status and the extremely low CD4+ count, less than 200/mm³, which indicates advancing HIV disease or AIDS. Also, we must keep in mind the patient's unprotected sexual practices, which represent a risk for further HIV transmission. Thus, the most clinically justified intervention would be to start antiretroviral therapy, both to slow the progression of the disease and lower the patient's viral load, reducing the risk of HIV transmission to his partners. 5) RESPONSE: Therefore, from a clinical standpoint and with the underlying data, (A) Antiretroviral therapy appears to be the most appropriate clinical intervention at this time.

**Facilitator** The consensus among the AI responses is that the most appropriate clinical intervention for the 25-year-old male patient, who presents with a white coating on his tongue, unintentional weight loss, and a positive HIV test with a low CD4+ T-lymphocyte count, is to initiate antiretroviral therapy (Option A). The white plaques on the tongue that cannot be scraped off, combined with the low CD4+ count and positive HIV status, suggest an opportunistic infection commonly seen in immune-compromised patients, such as oral hairy leukoplakia. However, the primary focus should be on managing the patient's HIV status due to the low CD4+ count, which indicates advancing HIV disease or AIDS. Antiretroviral therapy will slow the progression of the disease and lower the patient's viral load, reducing the risk of HIV transmission to his partners. Other treatment options, such as liquid nitrogen therapy, micafungin therapy, surgical excision of the tongue lesions, or systemic chemotherapy with liposomal doxorubicin, are not considered the most appropriate at this time as they do not address the underlying cause of the patient's symptoms.
